# Subunits of C1Q Are Associated With the Progression of Intermittent Claudication to Chronic Limb-Threatening Ischemia

**DOI:** 10.3389/fcvm.2022.864461

**Published:** 2022-04-01

**Authors:** Ziping Yao, Bihui Zhang, Guochen Niu, Ziguang Yan, Xiaoqiang Tong, Yinghua Zou, Min Yang

**Affiliations:** Department of Interventional Radiology and Vascular Surgery, Peking University First Hospital, Beijing, China

**Keywords:** peripheral artery disease, intermittent claudication, chronic limb-threatening ischemia, complement, mechanism, Bioinformatics

## Abstract

**Background:**

The pathophysiological mechanisms of intermittent claudication (IC) progression to chronic limb-threatening ischemia (CLTI) are still vague and which of patients with IC will become CLTI are unknown. This study aimed to investigate the key molecules and pathways mediating IC progression to CLTI by a quantitative bioinformatic analysis of a public RNA-sequencing database of patients with peripheral artery disease (PAD) to screen biomarkers discriminating IC and CLTI.

**Methods:**

The GSE120642 dataset was downloaded from the Gene Expression Omnibus (GEO) database. Differentially expressed genes (DEGs) between IC and CLTI tissues were analyzed using the “edgeR” packages of R. The Gene Ontology and the Kyoto Encyclopedia of Genes and Genomes enrichment analyses were performed to explore the functions of DEGs. Protein–protein interaction (PPI) networks were established by the Search Tool for the Retrieval of Interacting Genes (STRING) database and visualized by Cytoscape software. Hub genes were selected by plugin cytoHubba. Gene set enrichment analysis was performed and the receiver operating characteristic curves were used to evaluate the predictive values of hub genes.

**Results:**

A total of 137 upregulated and 21 downregulated DEGs were identified. Functional enrichment clustering analysis revealed a significant association between DEGs and the complement and coagulation cascade pathways. The PPI network was constructed with 155 nodes and 105 interactions. The most significantly enriched pathway was complement activation. C1QB, C1QA, C1QC, C4A, and C1R were identified and validated as hub genes due to the high degree of connectivity. The area under the curve values for the five hub genes were greater than 0.95, indicating high accuracy for discriminating IC and CLTI.

**Conclusion:**

The complement activation pathway is associated with IC progression to CLTI. C1QB, C1QA, C1QC, C4A, and C1R might serve as potential early biomarkers of CLTI.

## Introduction

Peripheral artery disease (PAD) is a limb manifestation of systemic atherosclerosis associated with an increased cardiovascular events and death (1, 2). Intermittent claudication (IC) and chronic limb-threatening ischemia (CLTI) are different severity stages of symptomatic PAD. However, only a minority of patients with IC will progress to CLTI, with an incidence ranging from 1.1 to 21% per 5 years (3, 4), suggesting that CLTI may be different from IC in etiologies (5, 6). Nevertheless, the pathophysiological mechanisms of IC progression to CLTI are still vague and which patients with IC will develop to CLTI are unknown.

Compared to IC, the prognosis of CLTI is much worse at the patient and limb levels (2). Early revascularization could reduce amputation and mortality (7). Thus, it is of great clinical relevance to identify patients with IC at high risk for deterioration to CLTI before the onset of gangrene or tissue loss. Although diabetes, hemodialysis, and a decrease in the ankle-brachial index have been identified as independent predictors of CLTI in long-term follow-up studies of patients with IC (3, 8), no methods that allow clinicians to estimate the risk of IC progressing to CLTI are currently available (7, 8). Biomarkers that help clinicians to stratify severities and predict prognosis could assist in the management of patients with PAD and can improve quality of life, reduce devastating morbidities, and extend longevity (9, 10).

In this study, we investigated the key molecules and pathways mediating IC progression to CLTI by a quantitative bioinformatic analysis of a public RNA-sequencing database of patients with PAD and estimated the value of these selected molecules as biomarkers (5).

## Methods

### Study Design and Data Collection

All the eligible datasets were screened from the Gene Expression Omnibus (GEO) database. The selection criteria were as follows: (1) inclusion of gene expression data of patients with IC and CLTI and (2) arrays containing a minimum of 10 tissue samples. One dataset (GSE120642) was finally included in this study according to the aforementioned screening criteria (5). GSE120642 included transcriptome information of gastrocnemius muscle samples from patients with IC and CLTI and healthy controls.

### Differentially Expressed Genes Identified by EdgeR

The series matrix files of datasets were downloaded from the GEO. The R package edgeR was utilized to find differentially expressed genes (DEGs) (11). The fold changes (FCs) in the expression of individual genes were calculated. Genes that met the specific cutoff criteria of *P* < 0.05 and |logFC| > 2.0 were defined as DEGs.

### Functional Annotation and Pathway Enrichment Analysis

To further reveal the functions of DEGs, the cluster profiler package in R software was used to conduct the Gene Ontology (GO) annotation and the Kyoto Encyclopedia of Genes and Genomes (KEGG) pathway enrichment analyses of DEGs. The GO terms comprised the following three divisions: biological process (BP), cellular component (CC), and molecular function (MF). *P* < 0.05 was regarded statistically significant.

The GO functional enrichment analysis and the KEGG pathway enrichment of the significant modules were conducted by using the Database for Annotation, Visualization, and Integrated Discovery (DAVID) (http://david.abcc.ncifcrf.gov/) online tool (12). *P* < 0.05 was set as the cutoff criteria.

### Protein–Protein Interaction Network Construction and Identification of Hub Genes

Based on the target genes identified, a protein–protein (PPI) interaction network was constructed by using the Search Tool for the Retrieval of Interacting Genes Database (STRING) (https://cn.string-db.org/) and visualization was performed by Cytoscape 3.9.0 (13). The confidence score was set as the highest (confidence score > 0.9). Moreover, the MCODE plugin in Cytoscape was utilized to filter the significant modules of core genes from the PPI network complex. The criteria were set as degree cutoff = 2, node score cutoff = 0.2, K-core = 2, and maximum depth = 100. Hub genes were identified using Cytohubba, a plugin of Cytoscape software.

### Gene Set Enrichment Analysis

To further investigate the biological pathways involved in the CLTI pathogenesis through the hub genes, gene set enrichment analysis (GESA) (http://software.broadinstitute.org/gsea/index.jsp) was used to assess related pathways and molecular mechanisms (14). The expression levels of hub genes were utilized as phenotype labels and the metric for ranking genes was set to Pearson's correlation. All the other basic and advanced fields were set to default. The KEGG gene set biological process database (c2.KEGG.v7.4) from the Molecular Signatures Database–MsigDB (http://www.broad.mit.edu/gsea/msigdb/index.jsp) was used for enrichment analysis. Enriched gene sets with a nominal *P* < 0.05 and a false discovery rate (FDR) of <0.05 were considered as statistically significant.

### Receiver Operating Characteristic Curve

Patients with PAD were classified into the IC and CLTI groups. The receiver operating characteristic (ROC) curves were generated to assess the predictive accuracy of discriminating IC and CLTI using the pROC package in the R language (15).

### Statistical Analysis

All the statistical analyses were performed using R software (version 4.1.2; https://www.r-project.org/). A value of *P* < 0.05 was considered statistically significant.

## Results

### Differentially Expressed Genes in IC and CLTI

**Figure 1:**
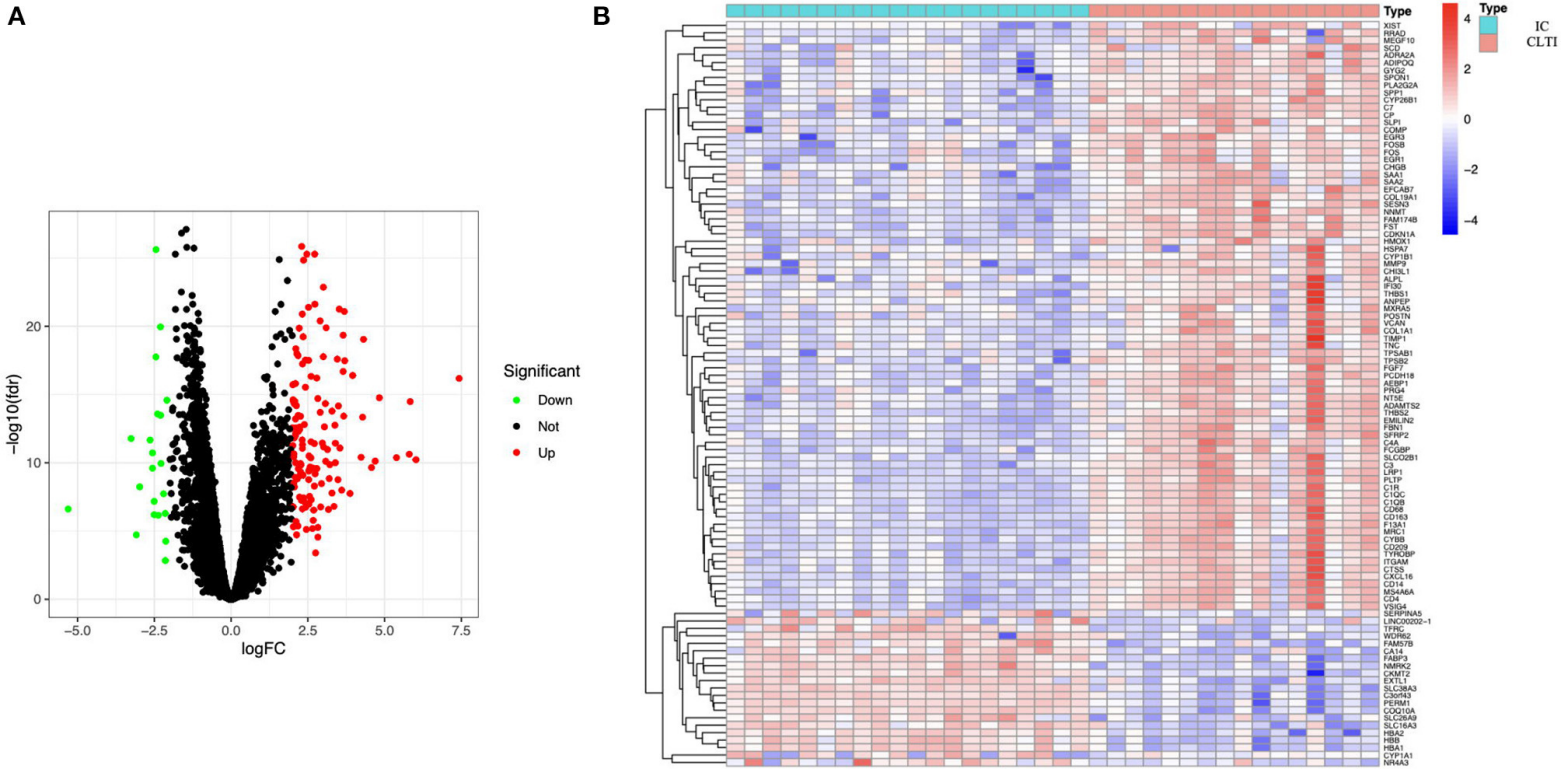
Samples from patients with IC and CLTI have different gene expression profiles. **(A)** Volcano plot revealed 137 upregulated intersecting genes and 21 downregulated genes between the IC and CLTI groups. **(B)** Heatmap of the top 50 upregulated genes and all the downregulated genes in GSE120642. IC, intermittent claudication; CLTI, chronic limb-threatening ischemia.

A total of 158 DEGs, 21 downregulated and 137 upregulated, were identified in CLTI samples compared with the IC samples (Figure 1A). The heatmap showed that these DEG patterns could distinguish IC and CLTI samples (Figure 1B).

### Gene Ontology and Kyoto Encyclopedia of Genes and Genomes Pathway Analyses

**Figure 2:**
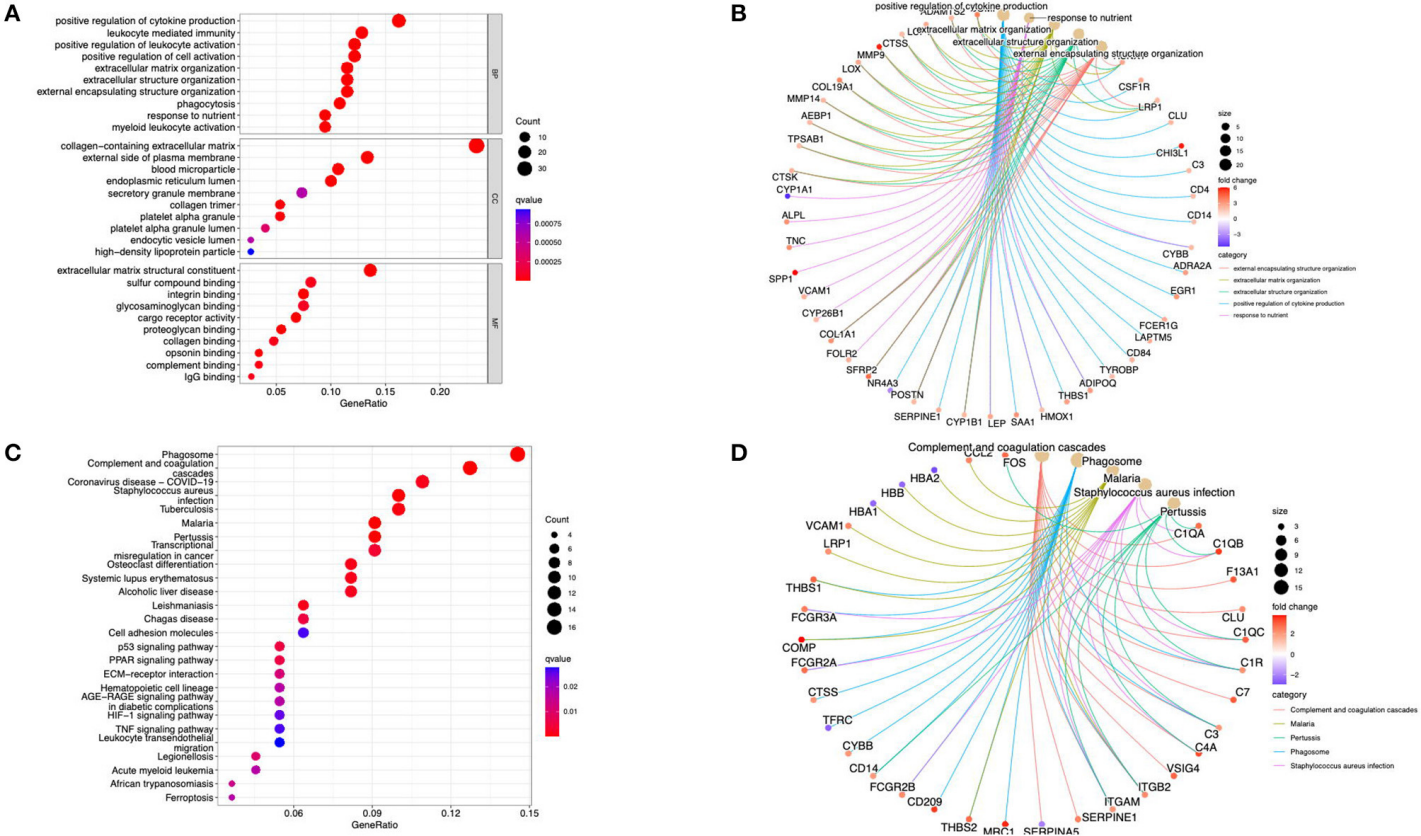
Functional enrichment analysis of the DEGs. **(A)** The GO terms in biological process, cellular component, and molecular function were performed for functional enrichment clustering analysis. **(B)** The cent plot of the GO analysis shows the pathway-gene network. **(C)** The KEGG pathway analysis was performed of DEGs. **(D)** The cent plot of the KEGG analysis shows the pathway-gene network. DEG, differentially expressed genes; GO, gene ontology (GO); KEGG, Kyoto Encyclopedia of Genes and Genomes.

The GO analysis and the KEGG pathway enrichments were performed using the cluster profiler package in R software to further explore the underlying biological information of these valid DEGs. These analyses demonstrated that the genes were mainly enriched in the positive regulation of cytokine production, response to nutrients, and extracellular matrix organization regarding the biological process. For the cellular component, the genes were mainly enriched in the collagen-containing extracellular matrix, blood microparticles, and the external side of the plasma membrane. Finally, regarding molecular function, the genes were mainly enriched in extracellular matrix structural constituents and proteoglycan binding (Figure 2A). The KEGG analysis revealed that DEGs were enriched in phagosome, complement, and coagulation cascades, *Staphylococcus aureus* infection, and tuberculosis (Figure 2C). In addition, the cent plot of the GO analysis and the KEGG signaling pathway shown in the pathway-gene network is shown in Figures 2B,D, respectively.

### Protein–Protein Interaction Network Construction and Functional Annotation of Module

**Figure 3:**
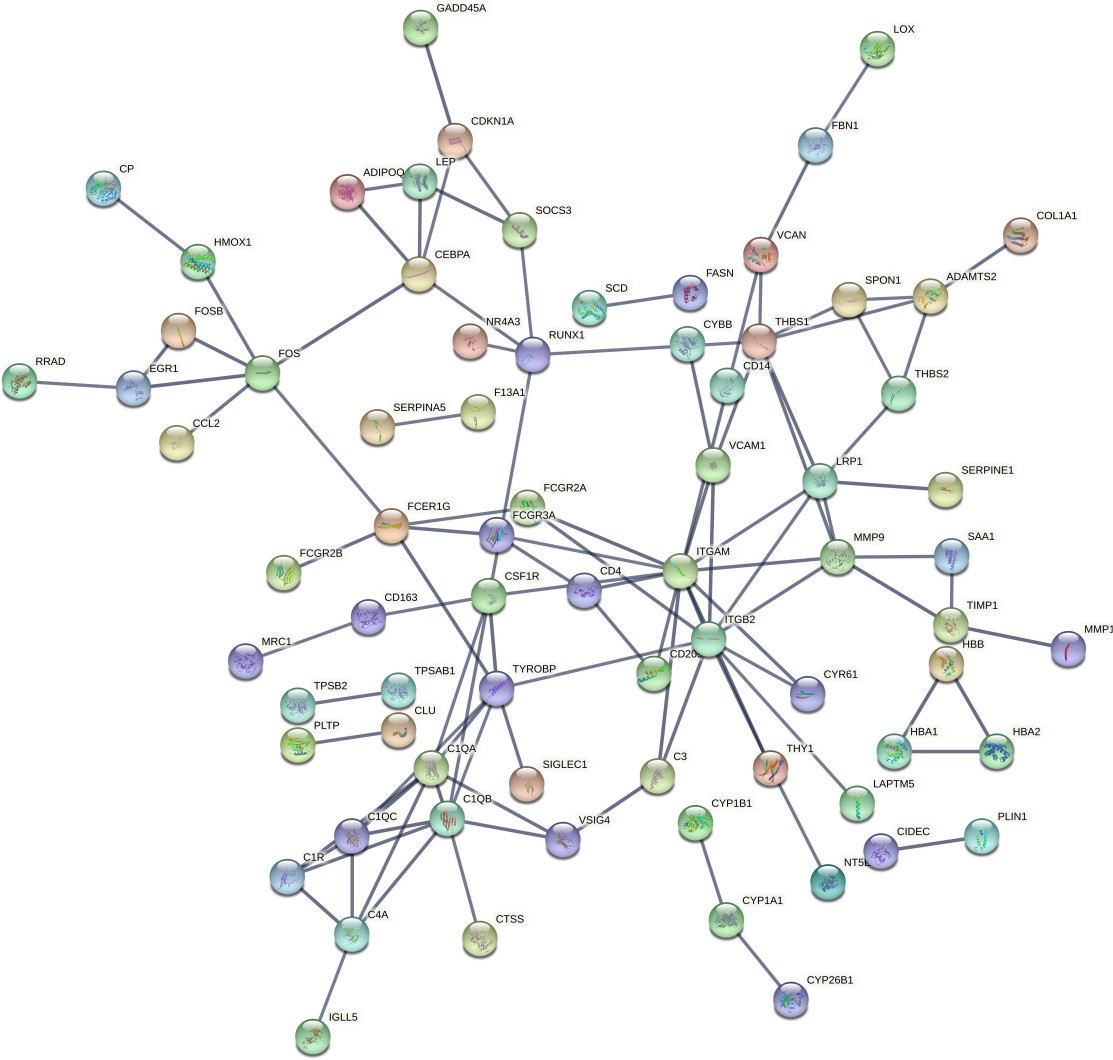
PPI network of DEGs constructed by the STRING. PPI, protein–protein interaction; DEG, differentially expressed genes; STRING, Search Tool for the Retrieval of Interacting Genes.

The PPI network of the DEGs was screened and constructed *via* the STRING and included 155 nodes (genes) and 105 edges (interactions). After removing the isolated and partially connected nodes, a complex network of DEGs was constructed, as shown in Figure 3.

**Figure 4:**
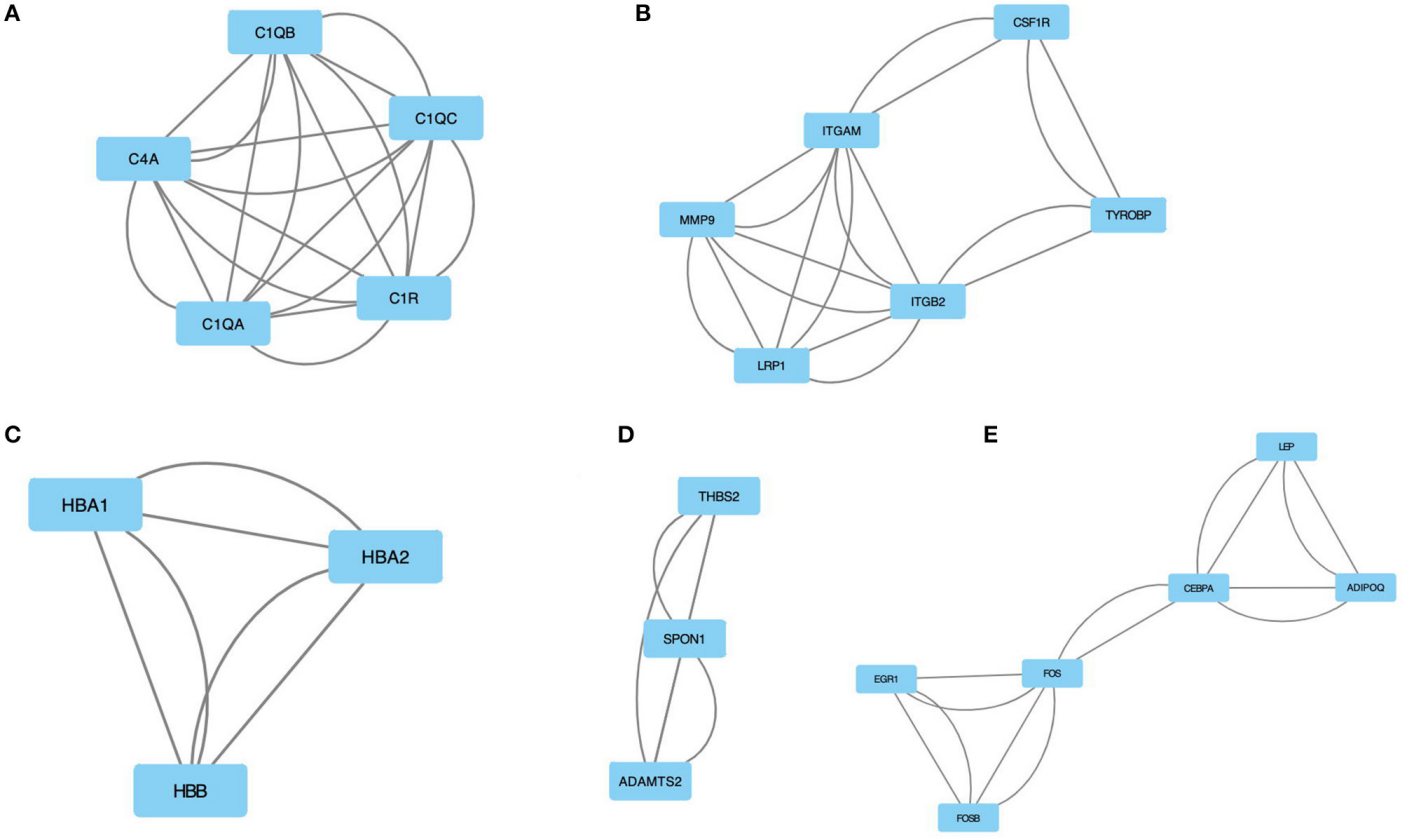
The module identified from the PPI network using the MCODE method is shown in this figure. **(A)** Module 1 with an MCODE score of 5. **(B)** Shows module 2 with an MCODE score of 3.6. **(C)** The module 3 with an MCODE score of 3. **(D)** The module 4 with an MCODE score of 3. **(E)** The module 5 with an MCODE score of 2.8. MCODE, molecular complex detection.

Using MCODE, five modules were retrieved from the PPI network constructed using DEGs (Figure 4). Module 1 includes 5 nodes and 20 edges with a cluster score (density multiplied by the number of members) of 5 (Figure 4A); module 2 includes 6 nodes and 18 edges with a cluster score of 3.6 (Figure 4B); modules 3 and 4 include 3 nodes and 6 edges with a cluster score of 3 (Figures 4C,D); module 5 includes 6 nodes and 14 edges with a cluster score of 2.8 (Figure 4E).

The GO and the KEGG pathway enrichments were performed for the most significant module to explore potential biological processes associated with CLTI. The GO analysis demonstrated that the module's gene function was associated with complement activation, serine-type endopeptidase activity, and blood microparticles (Table 1). The KEGG pathway enrichment analysis showed that this module is mainly enriched in *Staphylococcus aureus* infection, complement, and coagulation cascades (Table 2).

**Table 1:** Enrichment analysis of GO pathway of module 1.

**Category**	**Term**	**Count**	**%**	***P* value**	**FDR**
GOTERM_BP_DIRECT	GO:0006956~complement activation	5	100	6.72E−10	6.82E−09
GOTERM_BP_DIRECT	GO:0006958~complement activation, classical pathway	5	100	1.14E−09	6.82E−09
GOTERM_MF_DIRECT	GO:0004252~serine-type endopeptidase activity	5	100	5.09E−08	3.56E−07
GOTERM_BP_DIRECT	GO:0045087~innate immune response	5	100	4.24E−07	1.70E−06
GOTERM_BP_DIRECT	GO:0006508~proteolysis	5	100	7.77E−07	2.33E−06
GOTERM_CC_DIRECT	GO:0072562~blood microparticle	4	80	2.26E−06	2.26E−05
GOTERM_CC_DIRECT	GO:0005576~extracellular region	5	100	6.07E−05	3.04E−04
GOTERM_CC_DIRECT	GO:0005581~collagen trimer	3	60	1.50E−04	5.01E−04
GOTERM_CC_DIRECT	GO:0005602~complement component C1 complex	2	40	4.39E−04	0.001097364
GOTERM_CC_DIRECT	GO:0070062~extracellular exosome	5	100	5.65E−04	0.001130093
GOTERM_BP_DIRECT	GO:0006955~immune response	2	40	0.096585326	0.231804782

**Table 2:** Enrichment analysis of KEGG pathway of module 1.

**Category**	**Term**	**Count**	**%**	***P* value**	**FDR**
KEGG_PATHWAY	hsa05150:Staphylococcus aureus infection	5	100	3.39E−09	2.37E−08
KEGG_PATHWAY	hsa04610:Complement and coagulation cascades	5	100	9.27E−09	3.04E−08
KEGG_PATHWAY	hsa05133:Pertussis	5	100	1.30E−08	3.04E−08
KEGG_PATHWAY	hsa05322:Systemic lupus erythematosus	5	100	1.38E−07	2.41E−07

### Hub Genes Recognition and Validation

**Figure 5:**
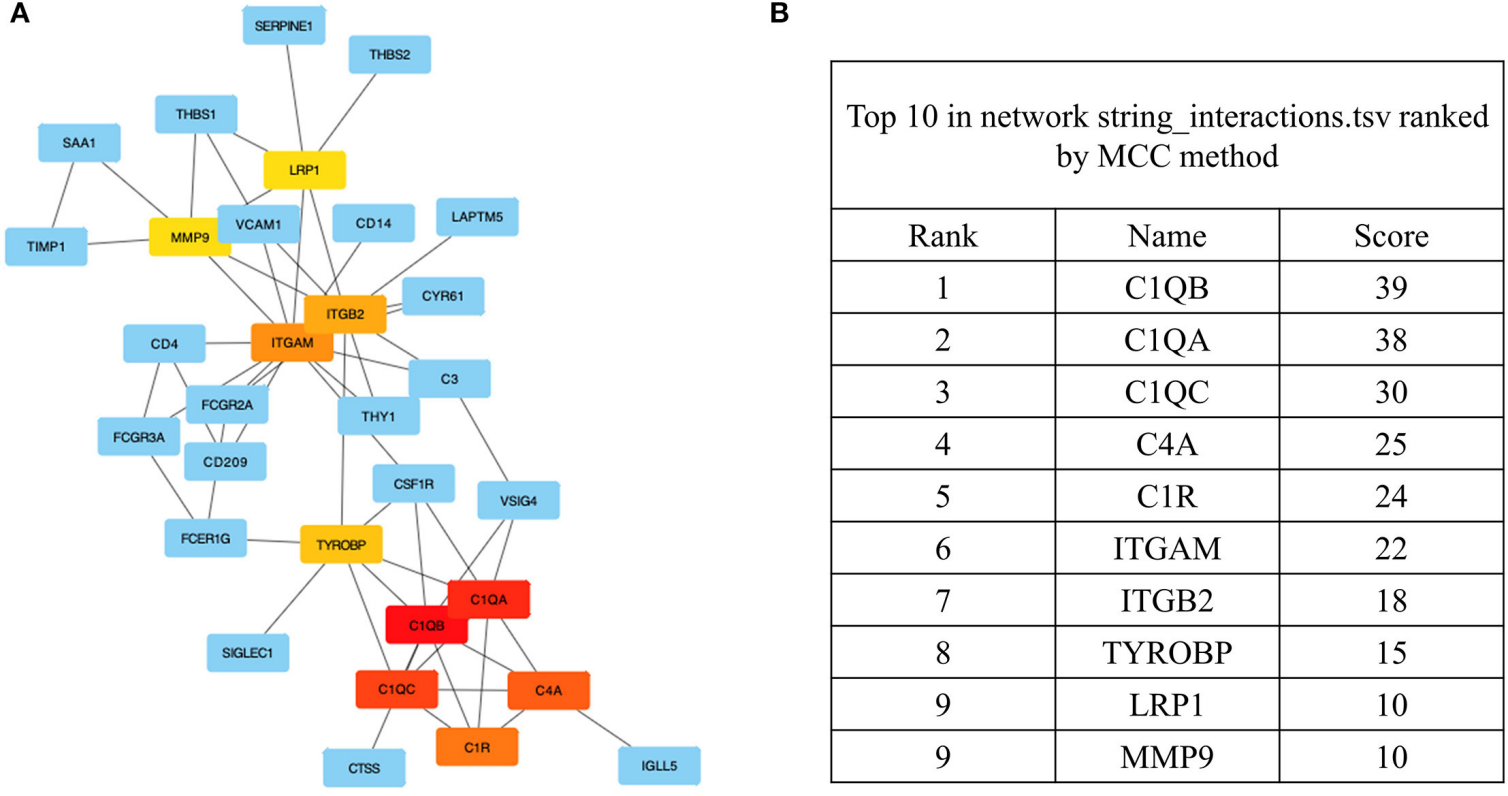
The identification of the hub genes. **(A)** Hub genes were identified from the PPI network using the MCC algorithm. Edges represent the protein–protein associations. The red nodes represent genes with high MCC scores, while the yellow nodes represent genes with low MCC scores. **(B)** The top 10 genes with a high MCC score are listed, and the top 5 were identified as hub genes. PPI, protein–protein interaction; MCC, maximal clique centrality.

Among the previously described DEGs, significant hub genes were identified by plug-in cytoHubba of Cytoscape using Maximal Clique Centrality (MCC) algorithm. All the gene codes and edges were calculated (Figure 5A). In total, five hub genes with the highest linkage degrees were identified, namely, C1QB (39), C1QA (38), C1QC (30), C4A (25), and C1R (24), which are all the crucial components of complement systems (Figure 5B).

**Figure 6:**
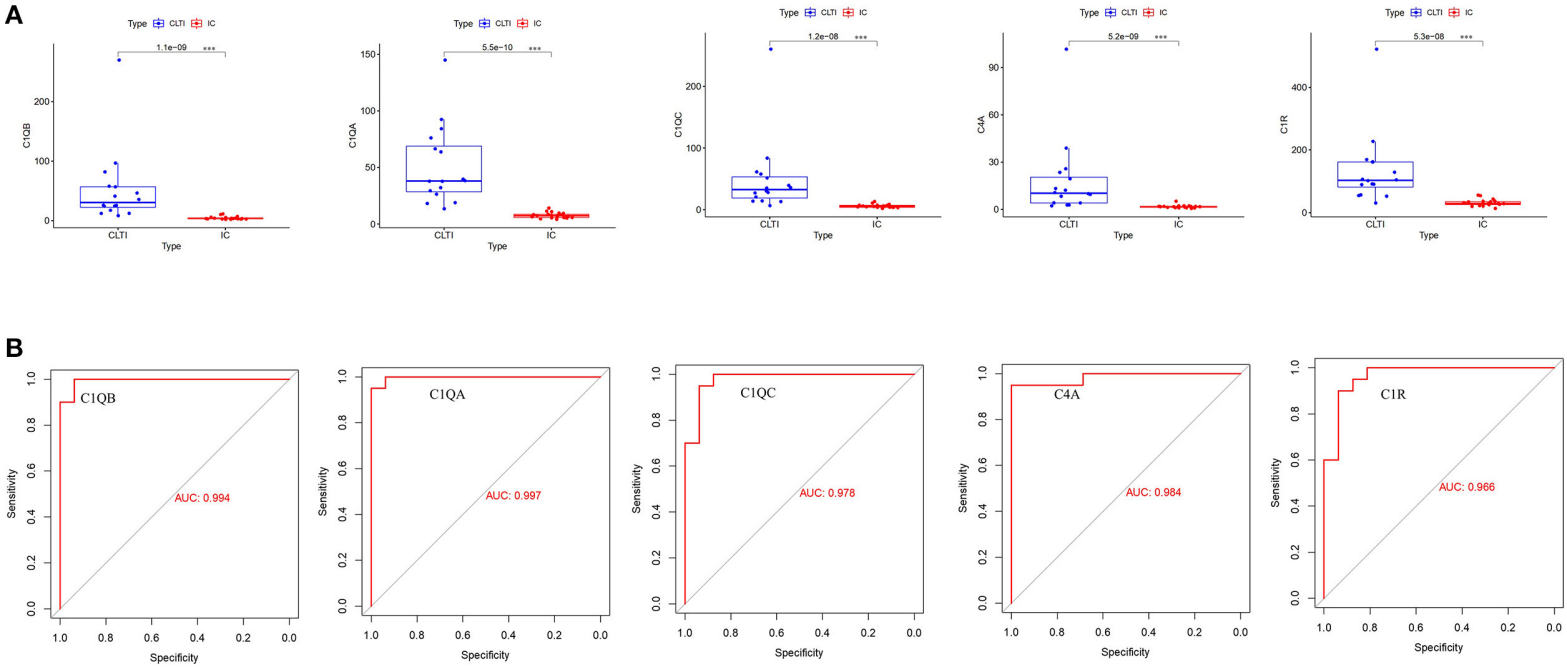
Validation of hub gene expression in GSE120642. **(A)** We checked the expression of hub genes in GSE120642, and their boxplot showed that they all had higher expression in CLTI tissues (***p < 0.001). **(B)** The receiver operating characteristic (ROC) curves of the hub genes were drawn to evaluate their accuracy in distinguishing IC and CLTI tissues. IC, intermittent claudication; CLTI, chronic limb-threatening ischemia; ROC, receiver operating characteristic.

All the hub gene expression levels were validated in IC and CLTI tissues. We unearthed five diagnostic genes and the data showed that they all had higher expression in CLTI tissues (*p* < 0.001) (Figure 6A). The receiver operating characteristic (ROC) curves were drawn to evaluate the capacity of hub genes to distinguish IC and CLTI tissues (Figure 6B). The areas under the curves (AUCs) of C1QB, C1QA, C1QC, C4A, and C1QR were 0.994, 0.997, 0.978, 0.984, and 0.966, respectively.

### Gene Set Enrichment Analysis

**Figure 7:**
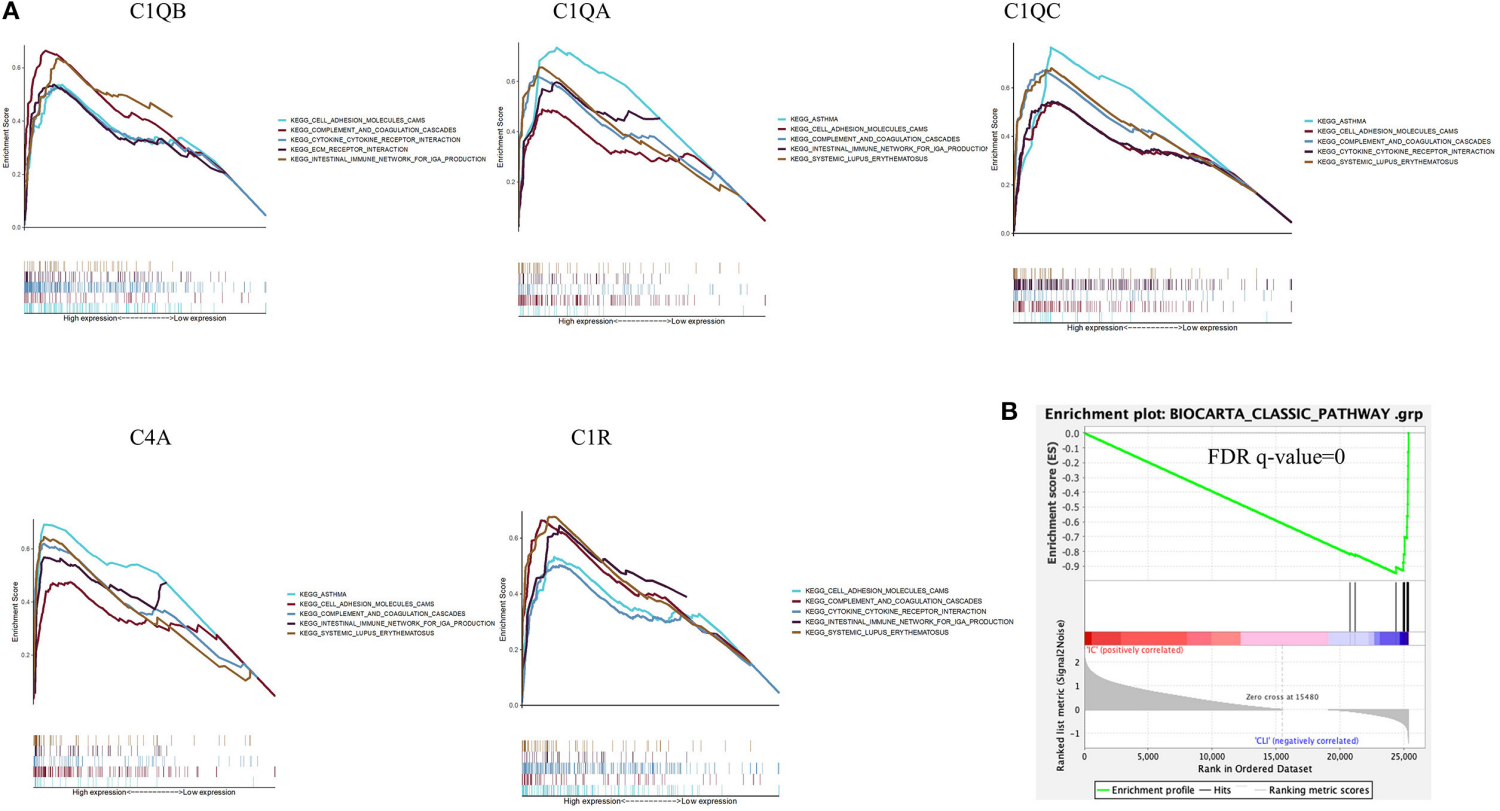
Gene set enrichment analysis. **(A)** A merged enrichment plot of C1QB, C1QA, C1QC, C4A, and C1R from gene set enrichment analysis, including enrichment score and gene sets. **(B)** The classical complement gene sets were significantly activated in CLTI tissues compared with IC tissues. IC, intermittent claudication; CLTI, chronic limb-threatening ischemia.

Among all the 176 predefined KEGG pathway gene sets, the complement and coagulation cascade pathway and cytokine–cytokine receptor interaction pathway were closely correlated with higher C1QB (C1QA, C1QC, C4A, and C1R) expression (Figure 7A).

Based on the reported biological functions of the selected hub genes, we further assessed classical complement pathways in patients with CLTI and found that the complement gene sets were significantly activated in CLTI samples compared with IC samples (Figure 7B).

## Discussion

This study analyzed gene expression to explore DEGs in the lower limb samples of IC and CLTI by bioinformatic approaches. Underlying biological pathway analyses and network construction were also performed. Finally, genes encoding subunits of the human complement system C1Q (C1QA, C1QB, and C1QC), C4A, and C1R were recognized as hub genes and can be promising biomarkers in discriminating CLTI and IC.

The distinct difference in prognosis makes it essential to investigate which part of patients with IC will progress to CLTI and the molecular biological mechanism of evolution (7). A prospective cohort study enrolling 1,107 *de-novo* patients with IC elucidated that diabetes and hemodialysis were independent predictors of CLTI. A history of cerebral infarction and femoropopliteal revascularization tended to increase CLTI (3). Another prospective study with 1,244 patients with IC documented that the ankle–brachial index and diabetes were associated with rest pain and ulceration (16). Plasma levels of soluble Tie2 and vascular endothelial growth factor (VEGF) were significantly increased in CLTI, exhibiting the potential to be novel biomarkers (17). CLTI was also independently associated with elevated levels of various cytokines compared with IC (18). Mechanistically, extensive skeletal muscle cell mitochondriopathy and fibrosis have been reported to distinguish CLTI from IC and may provide new targets for therapies and prediction (5, 6). The differences in IC and CLTI transcriptomes are also vital to identify the pathways that mediate the progression process of patients with IC. Complement activation was identified as the key pathway in both the GO analysis and the KEGG pathway enrichment analysis in this study, with subunits of C1Q, C4A, and C1R as the hub genes. The complement system is composed of over 30 proteins arranged in a proteolytic cascade ending in complement activation with the formation of the membrane attack complex (19). The complement system involves four pathways of activation (the classical, lectin, alternative, and extrinsic pathways) and plays a dual role in the genesis and development of atherosclerosis (20). The complement system could participate in atherosclerosis and vascular remodeling by several mechanisms, including an initial protective response that aims to clear cell debris and a potentially harmful role participating in leukocyte chemotaxis and cell activation and bridging innate and adaptive immunity (19). Complement components, including C5A, C3A, and C4, have been reported to be associated with cardiovascular diseases (21–23).

C1Q, together with C1R and C1S, forms the C1 complex that activates the complement system in the classical component pathway (24). C1Q is also found to mediate the enhancement of phagocytosis, regulation of cytokine production, and subsequent alteration in T-lymphocyte maturation in the absence of C1R and C1S (24). A recent study revealed that C1Q actively participates in primary hemostasis by promoting the arrest of bleeding (25). C1Q has potential proatherogenic and protective effects in atherosclerotic diseases. Several studies have shown that C1Q can recognize and clear apoptotic cells, which play a beneficial role (26–28). However, activation of the complement system by C1Q will exacerbate the later atherosclerotic process (29). A recent clinical study with 1,336 patients demonstrated that serum C1Q was associated with an increased risk of coronary artery disease and 1-year restenosis after revascularization (30). To the best of our knowledge, the role of C1Q in PAD is investigated for the first time, and all the subunits of C1Q (C1QA, C1QB, and C1QC) are associated with the progression of IC to CLTI in this study.

In comparison with the coronary heart disease, biomarkers in PAD remain largely exploratory at present (10). In the Atherosclerosis Risk in Communities (ARIC) study, the associations of high-sensitivity cardiac troponin T and natriuretic peptide NT-proBNP with incident PAD were examined among 12,288 middle-aged adults. In demographically adjusted models, the highest category of hs-cTnT and NT-proBNP showed ~8- and 10–20-fold higher risk of PAD and CLTI, respectively (31). A recent study determined that inflammatory and thrombotic biomarkers, including the neutrophil-to-lymphocyte ratio, monocyte-to-high-density lipoprotein-cholesterol ratio, fibrinogen-to-albumin ratio, and whole-blood viscosity, were elevated in PAD, while high-sensitive C reactive protein levels were not (32). Subunits of C1Q, C1R, and C4A could be used to discriminate CLTI and IC accurately with all the AUC values > 0.95 in this study. It remains to be seen whether they can be biomarkers in individuals.

This study had several limitations. First, the key pathways and hub genes identified in this study had not been validated in the laboratory experiments and clinical studies. Second, although complement activation and C1Q were found to be associated with the IC progression to CLTI, the definite mechanisms were unknown. Third, the molecules investigated were from gastrocnemius muscle samples. Whether the circulating levels of these molecules can be used as biomarkers is still unidentified. Further studies are needed on these aspects.

## Conclusion

This bioinformatic study elucidated that complement activation is the key pathway, and C1QB, C1QA, C1QC, C4A, and C1R are the hub genes in the progression of IC to CLTI. These genes can be diagnostic biomarkers discriminating CLTI and IC with excellent accuracies.

## Data Availability Statement

The datasets presented in this study can be found in online repositories. The names of the repository/repositories and accession number(s) can be found in the article/supplementary material.

## Author Contributions

ZY and BZ contributed in the data analysis and writing. GN and ZY contributed in data collection. XT contributed in study design. BZ, YZ, and MY also contributed in study design. All authors contributed to the article and approved the submitted version.

## Funding

This study was supported by the National Key R&D Program of China (Grant No. 2017YFC0109105), the Scientific Research Seed Fund of Peking University First Hospital (Grant No. 2018SF023), the Youth Clinical Research Project of Peking University First Hospital (Grant No. 2018CR16), and the Interdisciplinary Clinical Research Project of Peking University First Hospital (Grant No. 2018CR33).

## Conflict of Interest

The authors declare that the research was conducted in the absence of any commercial or financial relationships that could be construed as a potential conflict of interest.

## Publisher's Note

All claims expressed in this article are solely those of the authors and do not necessarily represent those of their affiliated organizations, or those of the publisher, the editors and the reviewers. Any product that may be evaluated in this article, or claim that may be made by its manufacturer, is not guaranteed or endorsed by the publisher.
